# Intranodal palisaded myofibroblastoma of the supraclavicular region in an adolescent with an HDAC2 variant: a case report

**DOI:** 10.3389/fmed.2026.1889299

**Published:** 2026-08-27

**Authors:** Yiyang Yu, Yiming Zhao, Lili Cao

**Affiliations:** 1 School of Clinical Medicine, Shandong Second Medical University, Weifang, Shandong, China; 2 Department of Oncology, Zibo Central Hospital, Zibo, Shandong, China

**Keywords:** benign mesenchymal tumor, cyclin D1, HDAC2, intranodal palisaded myofibroblastoma, supraclavicular lymph node

## Abstract

Intranodal palisaded myofibroblastoma (IPM) is a rare benign mesenchymal tumor of lymph nodes that most often arises in the inguinal region. We report an 18-year-old woman with an incidentally detected left supraclavicular mass. Imaging showed a well-circumscribed hypervascular lesion, and core biopsy demonstrated a spindle-cell neoplasm for which low-grade malignancy could not be excluded. Complete excision revealed an intranodal spindle-cell proliferation with nuclear palisading, hemorrhage, compressed peripheral lymphoid tissue, and hyalinized fibers. The morphology and immunophenotype supported IPM. Targeted sequencing identified an HDAC2 p.V12Sfs^*^8 variant, whereas no CTNNB1 variant was detected. Because no functional validation was performed, the biological significance of the HDAC2 variant remains uncertain. This case highlights IPM in an adolescent at an uncommon supraclavicular site and emphasizes careful pathological evaluation of spindle-cell lymph-node lesions.

## Introduction

Intranodal palisaded myofibroblastoma (IPM) is a rare, benign mesenchymal tumor originating from smooth muscle cells and myofibroblasts. Reported sites of occurrence include inguinal lymph nodes (1), submandibular lymph nodes (2), parotid lymph nodes (3), paraesophageal lymph nodes (4), axillary lymph nodes (5), paratracheal lymph nodes (6), retroperitoneal lymph nodes (7), cervical lymph nodes (8), and supraclavicular region lymph nodes (9). Given its extremely low incidence, clinicians have limited knowledge of IPM.

We searched PubMed and Crossref from database inception to 11 July 2026. The search combined “intranodal palisaded myofibroblastoma,” “intranodal hemorrhagic spindle-cell tumor,” or “palisaded myofibroblastoma” with anatomical terms for extra-inguinal sites. We also screened the reference lists of relevant reviews and case reports. Because previous literature recognizes supraclavicular IPM, we do not claim that the present tumor is the first at this site (8, 9).

We report an uncommon supraclavicular IPM in an 18-year-old woman and describe an HDAC2 variant of uncertain biological significance.

## Case description

**Figure 1:**
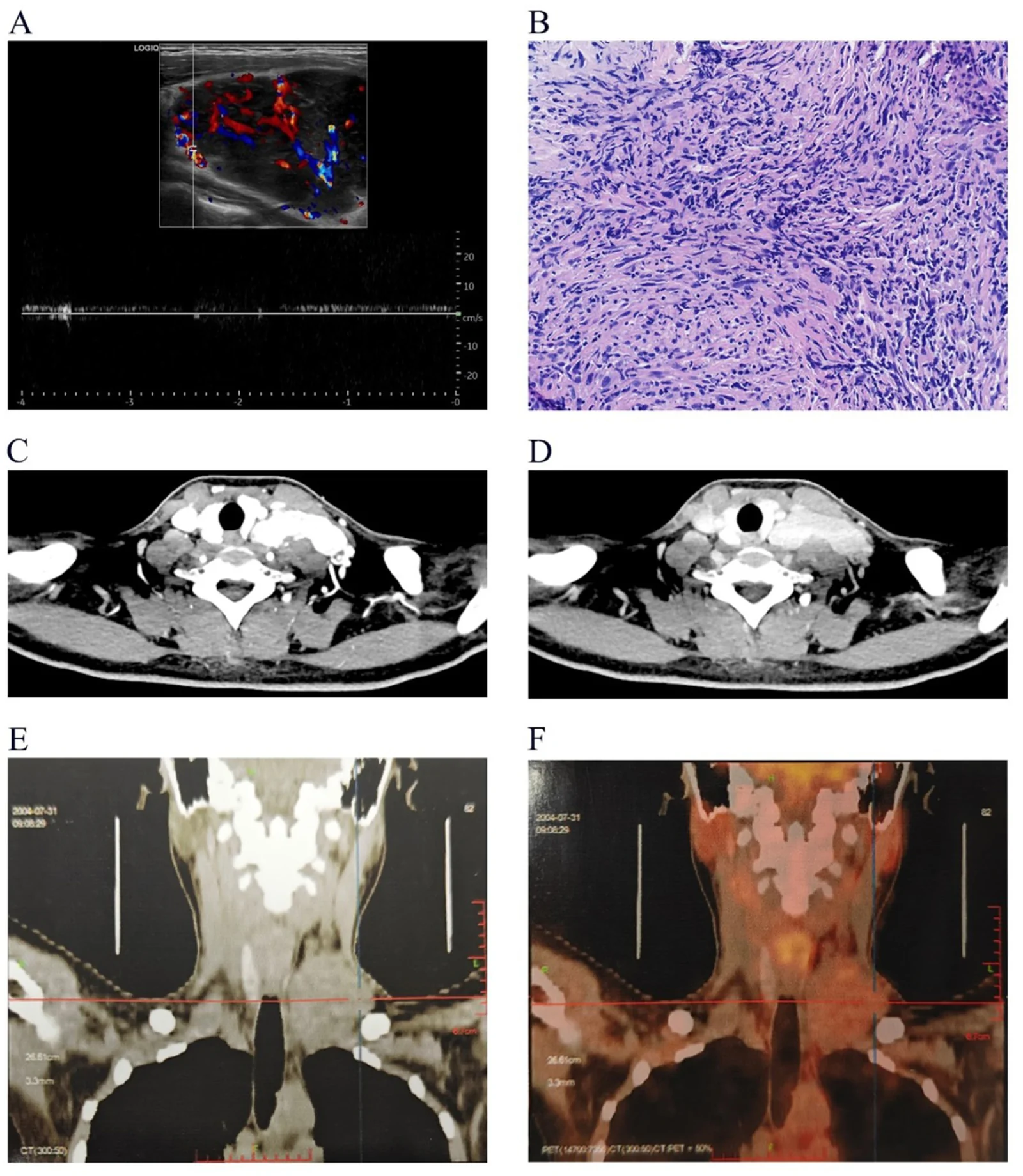
Preoperative imaging and core-biopsy findings. **(A)** Ultrasonography shows a well-circumscribed hypoechoic lesion with abundant internal vascularity. **(B)** Core biopsy shows spindle-cell proliferation. **(C, D)** Contrast-enhanced CT shows marked enhancement and internal vascular structures. **(E, F)** PET/CT shows heterogeneous FDG uptake (SUVmax, 1.9).

**Figure 2:**
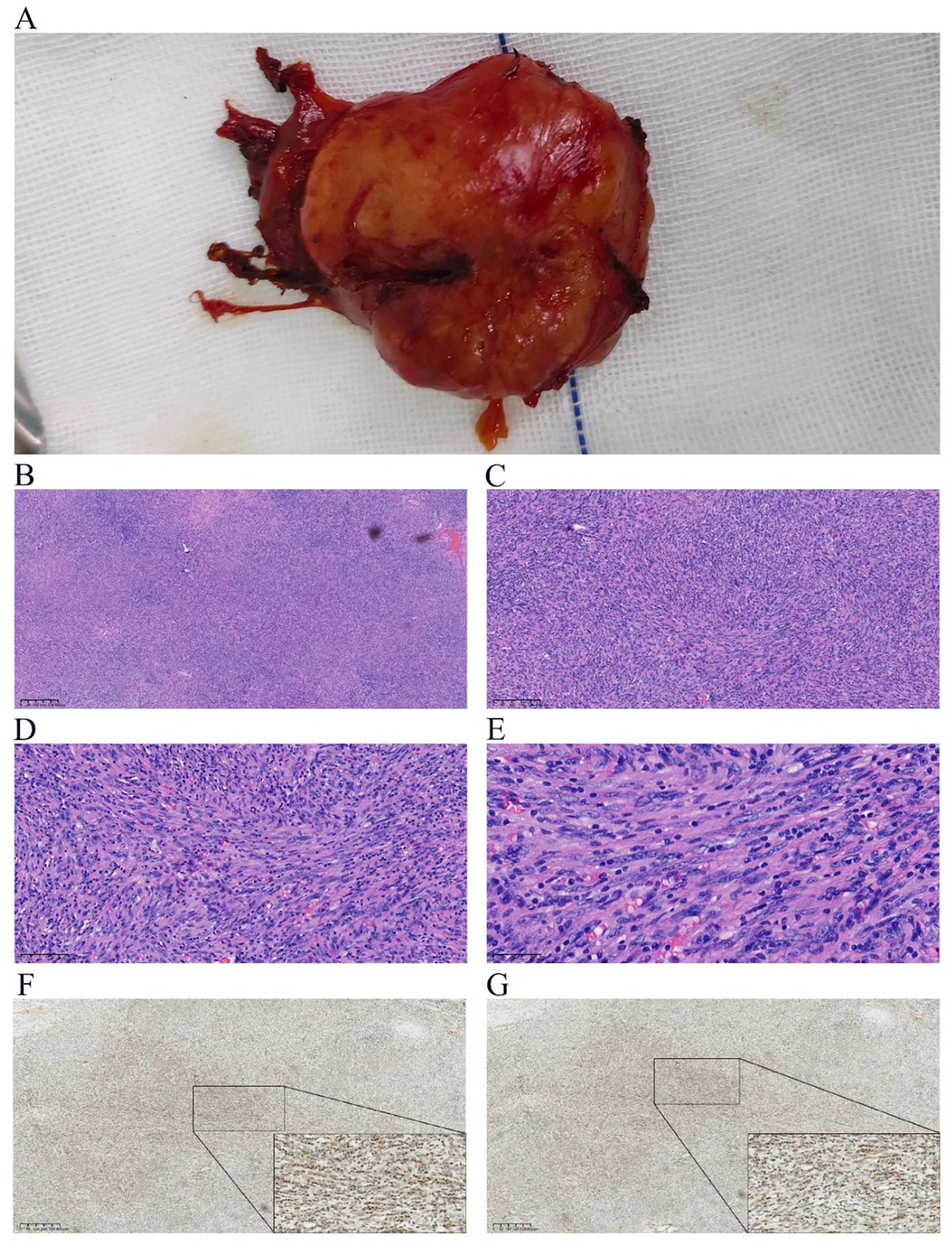
Operative and excision-pathology findings. **(A)** Gross appearance of the excised lesion. **(B–E)** Representative hematoxylin and eosin images at the stated magnifications. **(F, G)** The remaining panels show β-catenin and cyclin D1 immunohistochemistry.

**Figure 3:**
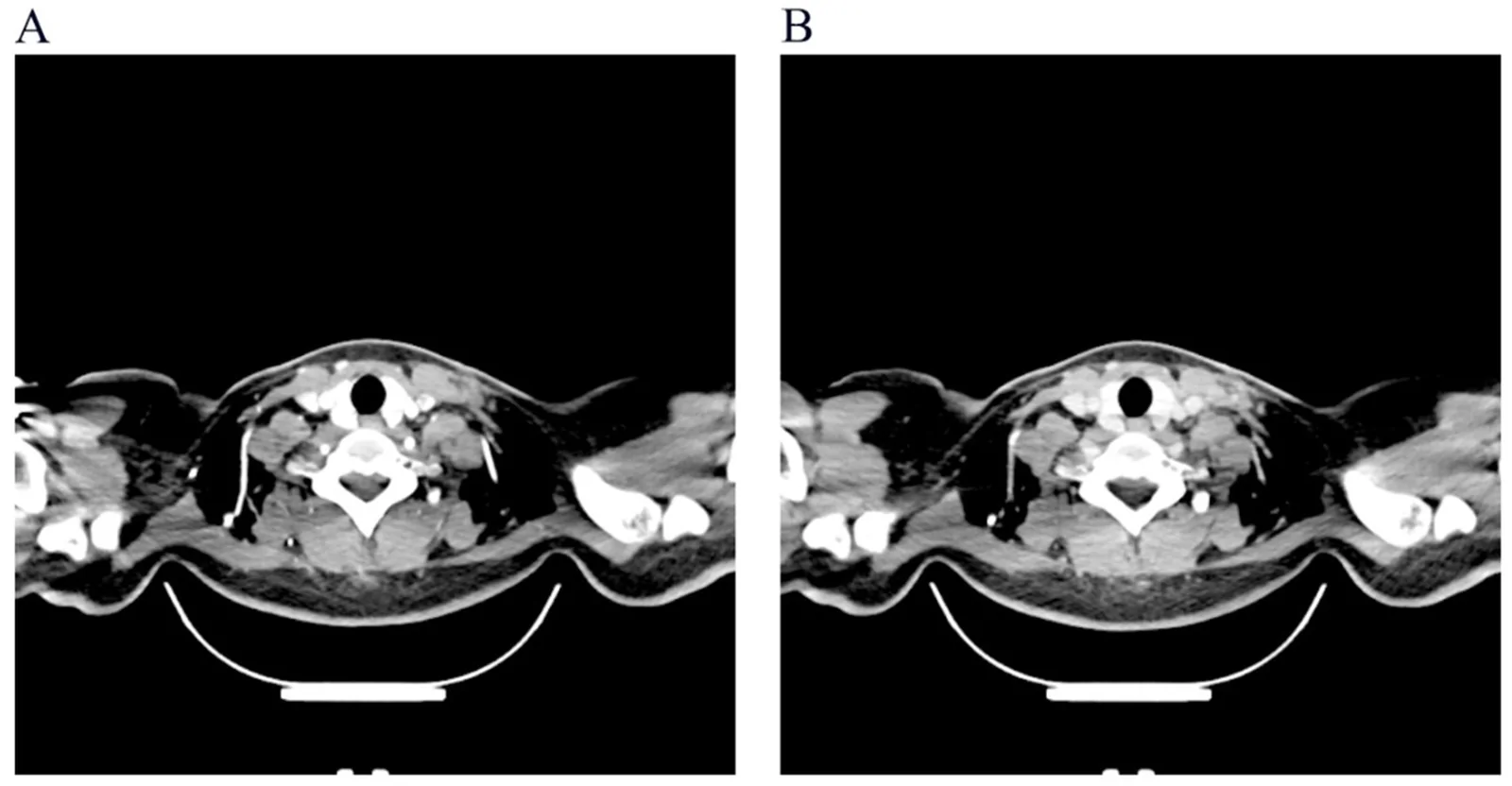
Representative contrast-enhanced images obtained during postoperative follow-up. **(A)** Arterial phase. **(B)** Venous phase.

The patient, an 18-year-old female, was admitted for the first time on June 19, 2024, due to “shortness of breath and chest tightness after activity for 1 month” and was diagnosed with viral pneumonia complicated by *Staphylococcus aureus* infection. During treatment, an asymptomatic mass was incidentally discovered in the left supraclavicular region. Ultrasound revealed a hypoechoic soft tissue mass in the left supraclavicular region, measuring approximately 5.9 cm × 4.0 cm × 2.1 cm, with well-defined borders and abundant internal vascular signals (Figure 1A). Needle biopsy pathology revealed spindle cell proliferation in the left supraclavicular mass, suggesting a spindle cell tumor, with the possibility of low-grade malignancy not excluded. Immunohistochemistry (IHC) demonstrated: Vimentin (+), SMA (–), Desmin (–), S-100 (+), CD34 (+), ERG (partial +), LCA (+), CD68 (+), CD3 (T cells +, spindle cells –), β-catenin (cytoplasmic +), MyoD1 (–), Myogenin (–), ALK (–), CD30 (–), CD21 (–), CD1a (–), CD20 (–), Ki-67 (+20%; Figure 1B). Neck CT revealed a mass of soft tissue density on the left side of the neck, medial to the left sternocleidomastoid muscle and posterior to the internal jugular vein, measuring approximately 5.3 cm × 2.4 cm × 5.1 cm. The mass exhibited well-defined borders with surrounding tissues, a CT value of about 43 Hu, and significant uniform enhancement on contrast-enhanced imaging. Multiple vascular images were seen within (Figures 1C, D) PET-CT demonstrated a soft tissue mass in the left supraclavicular region with moderate to high uneven uptake of FDG, measuring approximately 4.9 cm × 2.3 cm, SUVmax 1.9. A malignant lesion was suspected, with no evidence of distant metastasis observed (Figures 1E, F). To further elucidate the nature of the mass, a surgical excision biopsy was performed. Intraoperatively, the mass presented as gray-red, firm in texture, with an intact capsule, a rich blood supply, and a gray white, slightly gray-red cut surface (Figure 2A). Postoperative pathology revealed spindle cells arranged in a palisaded pattern, exhibiting oval-shaped vacuolated nuclei. Numerous lymphocytes and blood vessels were identified in the stroma, and hyalinized fibrous bundles were observed, in combination with immunohistochemistry suggested the diagnosis of intranodal palisaded myofibroblastoma. IHC results: CK (–), Desmin (partial +), CD3 (+), SMA (+), Ki-67 (+ 5%), CD34 (vascular +), STAT6 (–), CD68 (+), CD79α (focal +), CD21 (FDC +), CD20 (focal +), CD30 (–), ALK (–), CD15 (–), IgG (partial +), CD35 (–), CD23 (–), D2-40 (–), CD4 (+), S-100 (–), EBER (–; Figures 2B–G). Genetic testing revealed the following: HDAC2 p.V12Sfs^*^8 (Supplementary Table S1). The patient's postoperative CT scan showed no recurrence after XX months (Figures 3A, B).

## Discussion

IPM, also known as intranodal hemorrhagic spindle cell tumor with amianthoid fibers or solitary lymph node spindle cell tumor with myofibroblastic differentiation, is a benign mesenchymal tumor. This tumor is characterized by spindle cell proliferation, amianthoid fibers, hemorrhage within lymph nodes, and hemosiderin deposits, although its etiology and pathogenesis remain unclear (10). Reported cases indicate that the typical age range is between 45 and 55 years, with a slight male predominance and no significant racial differences (11). The tumor often occurs in the inguinal region. To our knowledge, this is an uncommon case of IPM occurring in the left supraclavicular area in an adolescent (Table 1).

**Table 1:** Previously reported cases of extra-inguinal IPM

Anatomic site	Representative literature	References
Submandibular	Several reports, including a 2024 case in a 31-year-old man	(2, 9)
Parotid	A recurrent case was reported in a 53-year-old man	(3)
Paraesophageal/mediastinal	Paraesophageal and right paratracheal tumors have been reported	(4, 6)
Axillary	Rare axillary tumors are documented in case reports and a 2025 review	(5, 9)
Retroperitoneal	Retroperitoneal IPM has been reported	(7, 9)
Cervical/supraclavicular	Reviews recognize cervical and supraclavicular presentations	(8, 9)
Present case	Left supraclavicular region; 18-year-old woman; HDAC2 variant of uncertain significance	Current report

In the pathological examination of this patient, the tumor was located at the center of the lymph node, surrounded by compressed residual lymphatic tissue that formed a pseudocapsule. The tumor comprised spindle cells, with abundant palisading areas evisible within. Marked hemorrhagic regions, extravasation of red blood cells, and hyalinized fibrous bundles were observed. These findings were consistent with the histological features of IPM, as summarized by Nguyen and Eltorky (11). Immunohistochemistry demonstrated positivity for mesenchymal and myogenic markers, including vimentin and SMA, while epithelial markers such as CD34 and Desmin were negative. Schwannomas also features palisading spindle cells, primarily occurring in peripheral nerve-innervated regions, with a very low likelihood of arising in lymph nodes. This patient exhibited no obvious neurological symptoms, and S-100 immunohistochemistry was negative. Inflammatory myofibroblastic tumors are mesenchymal neoplasms characterized by myofibroblastic proliferation within an inflammatory background. Immunohistochemistry typically demonstrates positive expression of vimentin and SMA, with ALK serving as its characteristic marker, although ALK-negative cases may exist. Histologically, spindle cells appear unorganized and are rarely arranged in palisades. Kaposi's sarcoma is characterized by significant vascular proliferation and abnormal vascular structures, accompanied by hemorrhage and hemosiderin deposition, and is often associated with immunosuppression. HHV-8 is a critical marker for diagnosis. Hodgkin's lymphoma typically presents as painless cervical lymphadenopathy in adolescents and is frequently associated with EBV infection, characterized by the presence of Reed-Sternberg cells. CD30 and CD15 are typically positive in this context.

The mechanisms underlying the development of IPM remain unclear. It has been suggested that IPM may originate from the vascular wall within lymph nodes, proliferating from myofibroblasts or mutated smooth muscle cells (12). Mutations in exon 3 of the CTNNB1 gene and codons affecting serine-threonine phosphorylation sites have been shown to disrupt the recognition site of β-catenin in the ubiquitin-dependent proteasome degradation pathway. This disruption results in the accumulation of downstream cyclin D1, which promotes cell transition from the G0 phase to the mitotic phase, thereby enhancing cell proliferation (13, 14). However, not all IPM cases in this study exhibited CTNNB1 mutations. In our reported case, no CTNNB1 gene mutation was found, however, HDAC2 p.V12Sfs^*^8 mutation was identified. This variant is a frameshift mutation in exon 1, causing a frameshift at the 12th position of the encoded protein, which results in a truncated protein or nonsense-mediated mRNA decay following the addition of eight extra amino acids. The loss of HDAC2 can lead to nuclear stabilization of β-catenin (15), which subsequently binds to the TCF/LEF transcription factor family, activating downstream target genes such as cyclin D1. However, these clues come from different biological backgrounds, and we did not conduct functional experiments afterwards, so we have no evidence to suggest the pathogenic role of HDAC2 in IPM.

In this report, we present an uncommon case of IPM occurring in the left supraclavicular area in an adolescent and provide a detailed description of its clinical data, alongside a brief discussion of the genetic testing results. Despite these findings, this report has several limitations. First, being a case report, it is subject to inherent bias. Secondly, the patient′s follow-up duration was short, and long-term follow-up data were unavailable. Finally, we have not yet thoroughly investigated the mechanisms underlying IPM's occurrence at this stage.

## Conclusions

We report an uncommon supraclavicular IPM in an 18-year-old woman, expanding the recognized age and anatomic spectrum of this benign tumor. Complete excision established the diagnosis. The HDAC2 p.V12Sfs^*^8 variant is a hypothesis-generating observation whose biological significance remains unknown without functional validation. Long-term follow-up is warranted because occasional recurrence has been reported.

## Data Availability

The original contributions presented in the study are included in the article/Supplementary material, further inquiries can be directed to the corresponding author.
