# Supplementary material for: Intranodal palisaded myofibroblastoma of the supraclavicular region in an adolescent with an HDAC2 variant: a case report
Source: Front Med (Lausanne). 2026 Aug 27;13:1889299. doi: 10.3389/fmed.2026.1889299 (PMC13560919; doi:10.3389/fmed.2026.1889299)
Supplement: Supplementary file 1 [file Table_1.docx]

Supplementary Table1 List of detected genes

AKT1(NM_001382430) CDH1(NM_004360) GNAQ(NM_002072) MSH2(NM_000251) RAD51C(NM_058216) AKT2(NM_001626) CDKN2B(NM_004936) GREM1(NM_013372) MSH6(NM_000179) RAD51D(NM_002878) AKT3(NM_005465) CDC73(NM_024529) GNAS(NM_000516) MTOR(NM_004958) RAF1(NM_002880) ALK(NM_004304) CHEK1(NM_00114122) HDAC2(NM_001527) MUTYH(NM_00148174) RB1(NM_000321) APC(NM_000038) CHEK2(NM_007194) H‑3‑3A(NM_002107) NBN(NM_002485) RET(NM_020975) AR(NM_000044) CTNNB1(NM_001904) H3C2(NM_003537) NF1(NM_001042492) RICTOR(NM_152756) ARAF(NM_001654) CTNNA1(NM_001903) H3C3(NM_003531) NOTCH1(NM_017617) RIT1(NM_006912) ATM(NM_000051) DDR2(NM_006182) HIF1A(NM_001530) NRAS(NM_002524) ROS1(NM_001378902) ARID1A(NM_006015) EGFR(NM_005228) HOXB13(NM_006361) NRG1(NM_013964) SDHA(NM_004168) ATR(NM_001184) EPCAM(NM_002354) HRAS(NM_005343) NTRK1(NM_002529) SDHB(NM_003000) ATRX(NM_000489) ERBB2(NM_004448) IGF1R(NM_000875) NTRK2(NM_006180) SDHC(NM_003001) AXL(NM_021913) ERBB3(NM_001982) IDH1(NM_005896) NTRK3(NM_001012338) SDHD(NM_003002) BCL2L11(NM_138621) ERCC3(NM_000122) IDH2(NM_002168) NFE2L2(NM_006164) SMAD4(NM_005359) BMPR1A(NM_004329) ESR1(NM_000125) JAK1(NM_002227) PALB2(NM_024675) SMARCA4(NM_003072) BRAF(NM_004333) FANCA(NM_000135) JAK2(NM_004972) PDGFRA(NM_006206) SMO(NM_005631) BRCA1(NM_007294) FBXW7(NM_001349798) JAK3(NM_000215) PDGFRB(NM_002609) STK11(NM_000455) BRCA2(NM_000059) FGFR1(NM_023110) KDR(NM_002253) PGR(NM_000926) TP53(NM_000546) BRIP1(NM_032043) FGFR2(NM_000141) KEAP1(NM_203500) PIK3CA(NM_006218) TSC1(NM_000368) BAP1(NM_004656) FGFR3(NM_000142) KIT(NM_000222) PMS2(NM_000535) TSC2(NM_000548) BARD1(NM_004465) FLT1(NM_002019) KRAS(NM_004985) POLD1(NM_002691) TERT(NM_198253) CCND1(NM_053056) FLT4(NM_182925) MAP2K1(NM_002755) POLE(NM_006231) TGFBR2(NM_003242) CCND2(NM_001759) FOXA1(NM_004496) MAP2K2(NM_030662) PTCH1(NM_000264) TSHR(NM_000369) CCND3(NM_001760) ABRAXAS1(NM_139076) MAPK1(NM_002745) PTEN(NM_000314) VEGFA(NM_003376) CCNE1(NM_001238) FANCL(NM_018062) MDM2(NM_002392) PAX8(NM_003466) VHL(NM_000551) CDK4(NM_001380) FH(NM_001413) MET(NM_000245) PIK3C2G(NM_001288772) CDK6(NM_001145306) FLCN(NM_144997) MLH1(NM_000249) RAD51B(NM_133510) CDKN1B(NM_004064) FLT3(NM_004119) MRE11(NM_005591) RAD54L(NM_003579) CDKN2A(NM_00077) GNA11(NM_002067) MYC(NM_002467) RAD51(NM_002875)
